# A Partner Hospital Intervention to Decrease Readmissions for Newborn Hyperbilirubinemia

**DOI:** 10.1097/pq9.0000000000000820

**Published:** 2025-06-03

**Authors:** Laura P. Chen, Elizabeth M. Goetz, Ann H. Allen, Daniel J. Sklansky, Kirsten Koffarnus, Kristin A. Shadman

**Affiliations:** From the * Department of Pediatrics, School of Medicine and Public Health, University of Wisconsin-Madison, Madison, Wis.; † Department of Pediatrics, American Family Children’s Hospital, Madison, Wis.

## Abstract

**Introduction:**

The 2022 American Academy of Pediatrics Clinical Practice Guideline revision for newborn hyperbilirubinemia raised thresholds for phototherapy initiation. Our global aim was to align care across 2 partner hospitals with the revised clinical practice guideline. Our aim was to decrease readmissions for phototherapy by 20% in 12 months.

**Methods:**

Using the model for improvement, a stakeholder team conducted this quality improvement initiative at our state’s largest birthing hospital and partner pediatric hospital. We collected baseline data from January to August 2022 and implementation data from September 2022 to February 2024. We included newborns 14 days or younger readmitted to the pediatric hospital general ward for phototherapy. Interventions included provider education, local clinical guidelines, and electronic medical record updates. Outcome measures of count and rate of monthly readmissions were tracked on a C chart and U chart, respectively. The process measure of time between occurrence of subthreshold phototherapy initiation was tracked on a t-chart. The balancing measure of the length of stay was analyzed on an XbarS chart. We assessed special cause variation using established statistical process control chart rules.

**Results:**

A total of 10,620 deliveries occurred, with 104 readmissions for hyperbilirubinemia. The mean count of monthly readmissions decreased from 5.8 to 2.4 from the baseline to the implementation period; the rate of monthly readmissions decreased from 1.4% to 0.6%. Mean days between the occurrence of subthreshold phototherapy initiation increased from 15.5 to 62.5 days. The average length of stay remained at 21.5 hours.

**Conclusions:**

This partner hospital initiative significantly decreased newborn hyperbilirubinemia readmissions.

## INTRODUCTION

In August 2022, the American Academy of Pediatrics (AAP) released a revised clinical practice guideline (CPG) for hyperbilirubinemia management of newborns born at 35 weeks gestational age or greater.^1^ Among the salient changes in the 2022 CPG was specific criteria for newborns to qualify for home phototherapy, along with higher thresholds for initiation of phototherapy. The rationale for raising the phototherapy thresholds was based on research demonstrating bilirubin neurotoxicity levels are much higher than the prior 2004 exchange transfusion thresholds.^2,3^ In addition, there is a potential correlation between newborn phototherapy and increased rates of epilepsy and infantile cancer.^4–7^

The revised CPG represented a significant practice change for many providers. Prior studies have demonstrated that provider utilization, adherence, and subsequent clinical outcomes of CPGs can be variable.^8,9^ Before the release of the 2022 revised CPG, there was a national increase in inpatient phototherapy use despite no change in the proportion of newborns with a diagnosis of jaundice.^10^ Opportunities to decrease inpatient phototherapy are important because it can impact caregiver-newborn bonding and caregiver mental health,^11,12^ influence the future perception of child vulnerability,^13–15^ and alter plasma metabolites.^16^ Furthermore, opportunities to decrease unnecessary admissions are critical in an era when pediatric hospital capacity is declining, and hospitals are often at capacity.^17^

The global goal of this quality improvement (QI) initiative was to align the management of newborns with hyperbilirubinemia across 2 collaborative partner hospitals (a birthing and nonbirthing pediatric hospital) with the 2022 CPG. We hypothesized that systematically implementing the increased phototherapy thresholds would lead to a decrease in readmissions. Our aim was to decrease newborn phototherapy readmissions by 20% in 12 months.

## METHODS

### Context and Study Design

This QI initiative was conducted at two partner hospitals within a Midwestern health system: a birthing and nonbirthing, freestanding pediatric hospital. The birthing hospital is our state’s largest birthing hospital, with almost 5,000 deliveries per year and a level III neonatal intensive care unit (NICU). The 111-bed pediatric hospital is a freestanding, tertiary care, university-affiliated children’s hospital with a level IV NICU. Hyperbilirubinemia readmissions are admitted to the general care unit of the pediatric hospital. Using the Institute for Healthcare Improvement Model for Improvement,^18^ a multidisciplinary stakeholder team from both hospitals gathered to design and implement this work. This team included pediatric hospitalists, newborn hospitalists, general pediatricians, nurses, pharmacy, laboratory medicine, and nutrition leaders. In October 2022, the local IRB determined this QI initiative did not constitute human subjects research and was thus exempt from full institutional review board review.

### Data Collection

Data were collected by retrospective chart review via Epic electronic health record (EHR) (Verona, Wis.) as part of the Learning and Implementing Guidelines for Hyperbilirubinemia Treatment QI collaborative led by the AAP Pediatric and Acute Critical Care Quality Network. We included newborns 0–14 days of age born at 35 weeks or greater gestational age and admitted to the pediatric hospital for the primary purpose of phototherapy. Newborns admitted to the NICU or pediatric intensive care unit were excluded as their primary admission diagnosis was not typically hyperbilirubinemia. Demographic data were collected, including newborn sex, self-reported race, delivery method, and gestational age. Laboratory data were collected as descriptive patient characteristics, including Direct Antiglobulin Test (DAT) status, whether there was concern for DAT-negative hemolysis, whether Glucose-6-phosphate dehydrogenase (G6PD) testing was obtained, and whether inappropriate rebound bilirubin was obtained (defined as rebound bilirubin obtained <18 h after phototherapy discontinuation for newborns without concerns for hemolysis who started phototherapy at <48 h of age). We collected baseline data from January 2022 to August 2022, and implementation data from September 2022 to February 2024.

### Measures

The primary outcome measures were the count of monthly readmissions and the rate of monthly readmissions for newborn hyperbilirubinemia to general care at our pediatric hospital. The rate of monthly readmission was calculated by dividing the monthly readmissions to the pediatric hospital by the number of births each month at our birthing hospital. The primary process measure was the time between occurrences of subthreshold phototherapy initiation, defined as starting phototherapy at ≥0.3 mg/dL below the AAP threshold. Phototherapy thresholds from the 2004 CPG were utilized for patients admitted from January 2022 to July 2022, and phototherapy thresholds from the 2022 CPG were applied to patients admitted after August 2022. A balancing measure of the length of stay (LOS) in hours assessed the impact of higher phototherapy thresholds in the 2022 CPG that may lead to admission at higher bilirubin levels and longer duration of phototherapy treatment.

### Analysis

The primary outcome measure of the count of monthly readmissions was analyzed on a statistical process control (SPC) C chart, and the rate of monthly readmissions was analyzed on an SPC U chart. The primary process measure of the occurrence of subthreshold phototherapy initiation was analyzed on an SPC t-chart using QI Charts (version 2.0.23; Performance Improvement Products, Austin, Tex.) and Microsoft Excel (version 16.79; Microsoft Corporation, Redmond, Wash.). LOS was analyzed on an XbarS chart. Improvement and assessment of special cause variation was evaluated using established SPC chart rules.^18^ Baseline and implementation demographics, DAT status, G6PD testing, and inappropriate rebound bilirubin obtainment were compared using a chi-square test, except gestational age was compared using an unpaired *t* test.

### Interventions

**Fig. 1.**
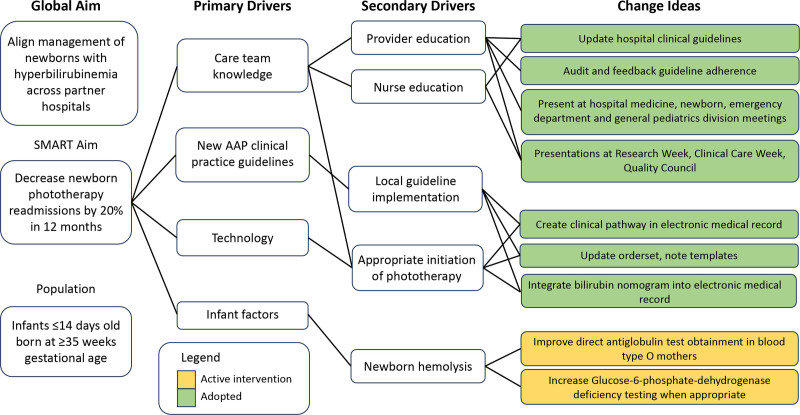
Key driver diagram demonstrating global aim, primary and secondary drivers, and change ideas to achieve the aim.

By applying improvement theory, a key driver diagram was developed to identify potential interventions to decrease newborn hyperbilirubinemia readmissions (Fig. 1).

#### Care Team Knowledge

Provider and nurse education were essential to disseminating the new AAP CPG amongst care team members at both hospitals. Several local leaders provided education via division meetings, including the Divisions of Hospital Medicine, Emergency Medicine, General Pediatrics, and the Newborn Nursery. Education in larger group venues occurred with department-wide presentations at Research Week in Spring 2023 and Clinical Care Week in Fall 2023, which were shared among providers from both the birthing and pediatric hospitals. Updated local clinical guidelines and EHR changes from both hospitals were shared with pediatric residents via an email listserv. We shared data from this QI project in Spring 2023 and Fall 2023 with our pediatric hospital Quality Care Council in a monthly meeting to share systemwide QI work.

At both hospitals, the existing institutional CPGs for Neonatal Jaundice were updated utilizing the AAP CPG by stakeholders, including physicians, nurses, pharmacy, laboratory medicine, and nutrition staff. The institutional CPGs were updated in March 2023 at the birthing hospital and in April 2023 at the pediatric hospital. As part of the improvement process, there was ongoing auditing and provider feedback on adherence to the new CPG at division meetings and the pediatric hospital Quality Care Council.

#### Technology

To facilitate the appropriate initiation of phototherapy using the new CPG, several EHR updates were made. In December 2022 at the pediatric hospital and January 2023 at the birthing hospital, the 2022 nomogram was integrated under a bilirubin activity tab. The bilirubin activity tab automatically displays for patients 7 days or younger, with the ability to access the tab for patients 8–15 days of age in an additional menu. On the bilirubin tab, transcutaneous and serum bilirubin levels are automatically plotted on the nomogram with the 2022 CPG phototherapy and exchange transfusion thresholds. In addition, the EHR note templates were updated at both hospitals. In December 2022, a smart phrase was created at the birthing hospital that incorporated the change in bilirubin level, presence or absence of neurotoxicity risk factors, and table with AAP follow-up recommendation. In October 2023, note templates for admission and discharge were created at the pediatric hospital to incorporate the updated AAP CPG.

In May 2023, at our pediatric hospital, an interactive clinical pathway was created within our EHR in partnership with AgileMD, a third-party vendor. The clinical pathway consists of an algorithm with actionable workflows, such as placing orders or visualizing pop-up windows with additional background information.

Also in May 2023, the EHR order set for neonatal jaundice at our pediatric hospital was updated to standardize phototherapy administration. Before the update, all newborns admitted to the pediatric hospital general care floor were placed on 2 overhead phototherapy lights at high-intensity settings and a bilirubin blanket. For low-risk newborns, the new order recommends 1 overhead light at a high-intensity setting and 1 bilirubin blanket. For newborns with concern for hemolysis or near escalation of care threshold, the order recommends consideration of 2 overhead lights and 1 bilirubin blanket. We performed provider and nurse education when these updates were made.

#### Newborn Factors

To decrease readmissions for phototherapy, we sought to improve the identification of newborns meeting phototherapy criteria during their birth hospitalization. At the birthing hospital, we wanted to improve the identification of newborns at risk for hemolysis secondary to ABO incompatibility. Before the 2022 CPG release, DAT was obtained on newborns with maternal blood type O and high-intermediate risk bilirubin levels. In August 2022, we changed this process so that DAT was obtained from cord blood on all newborns with maternal blood type O with bilirubin level >6.5 at 24 hours old. In addition, with the 2022 CPG’s increased focus on G6PD deficiency, our pediatric hospital’s institutional CPG was updated to consider G6PD screening if total serum bilirubin was not decreasing as expected with phototherapy or there was a concern for DAT-negative hemolysis.

## RESULTS

A total of 10,620 deliveries occurred at the birthing hospital during the 26-month study period. The overall births remained similar throughout the study period, with a mean of 401 births per month in the baseline period and 412 births per month in the implementation period. A total of 104 patients were readmitted to the pediatric hospital with hyperbilirubinemia. No demographic statistical differences existed between the baseline and implementation populations (Table 1). Readmitted patients were 60.6% male and 70.2% White, with an average gestational age of 38 weeks. The majority were born by vaginal delivery. Inappropriate rebound bilirubin level occurred in 16 (15.4%) patients, with 8.2% in the baseline period and 21.8% in the implementation period (*P* = 0.054) (Table 1).

**Table 1.** Demographics

	Total (n = 104), n (%)	Baseline (n = 49), n (%)	Implementation (n = 55), n (%)	*P*
Infant sex				0.083
Male	63 (60.6)	34 (69.4)	29 (52.7)	
Female	41 (39.4)	15 (30.6)	26 (47.3)	
**Infant race**				0.105
White	73 (70.2)	37 (75.5)	36 (65.5)	
Asian	7 (6.7)	1 (2)	6 (10.9)	
Black or African American	5 (4.8)	1 (2)	4 (7.3)	
More than 1 race	9 (8.7)	3 (6.1)	6 (10.9)	
Not listed	10 (9.6)	7 (14.3)	3 (5.5)	
Average gestational age (weeks)	38.2	38.3	38.2	0.563
Delivery method				0.163
Vaginal	93 (89.4)	46 (93.9)	47 (85.5)	
Cesarean section	11 (10.6)	3 (6.1)	8 (14.5)	
DAT status				0.079
Positive due to ABO or Rh incompatibility	15 (14.4)	6 (12.2)	9 (16.4)	
Positive due to passive transfer from Rhogam	1 (1)	1 (2)	0 (0)	
Negative	51 (49)	19 (38.8)	32 (58.2)	
Unknown or not tested	37 (35.6)	23 (46.9)	14 (25.5)	
Inappropriate rebound bilirubin	16 (15.4)	4 (8.2)	12 (21.8)	0.054

In the implementation period, G6PD testing was obtained on 1 of 3 newborns for whom we were concerned about DAT-negative hemolysis. No newborns in the baseline period exhibited concern for DAT-negative hemolysis.

**Fig. 2.**
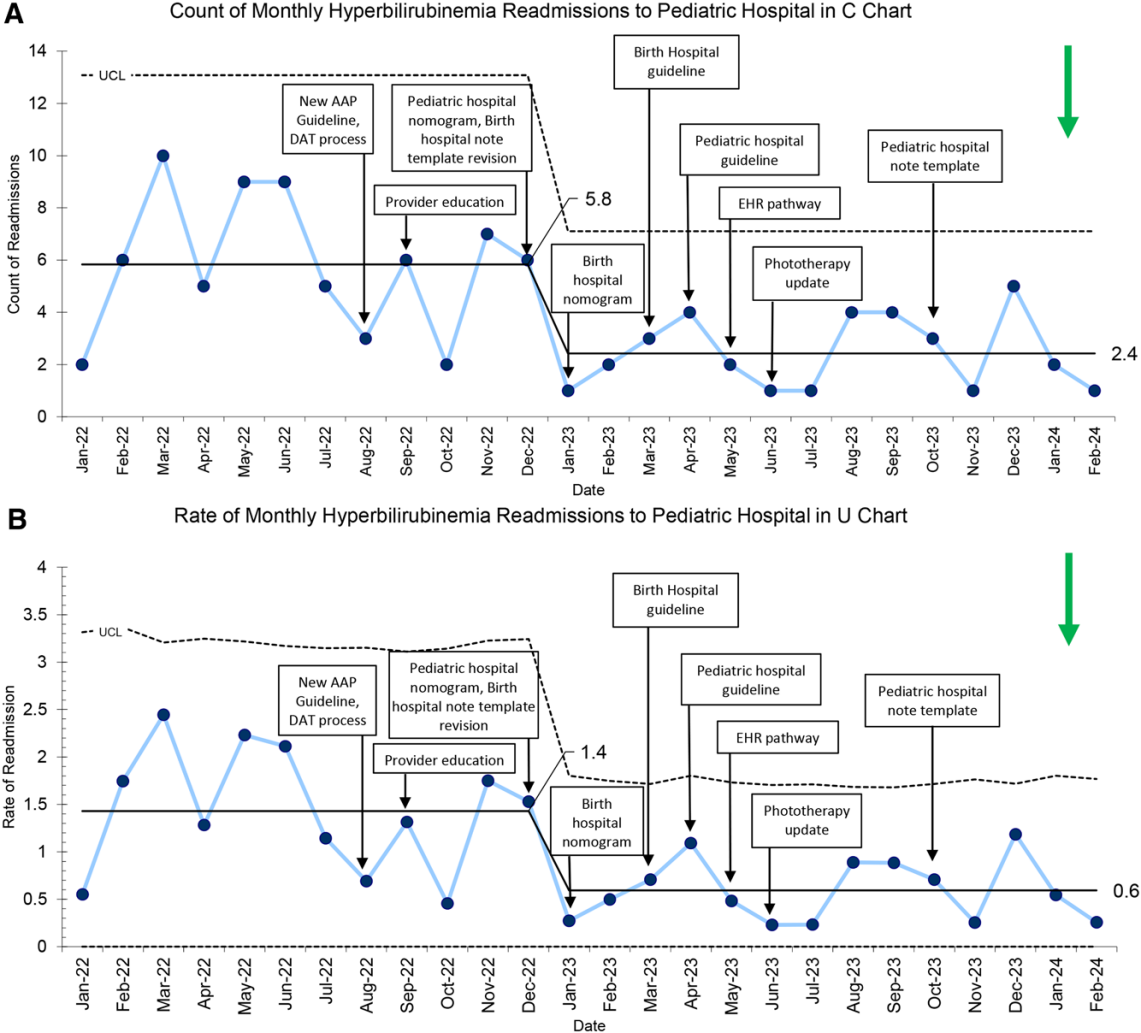
Count and rate of hyperbilirubinemia readmissions. A, SPC C chart of count of monthly hyperbilirubinemia readmissions to pediatric hospital with annotated interventions at birthing and nonbirthing pediatric hospital. The mean number of readmissions decreased from 5.8 to 2.4 patients per month. B, SPC U chart of rate of monthly hyperbilirubinemia readmissions to pediatric hospital with annotated interventions. The rate of readmissions decreased from 1.4% to 0.6% per month. UCL, upper control limit.

Before the release of the 2022 AAP CPG, the mean number of readmissions for newborn hyperbilirubinemia was 5.8 patients per month. The primary outcome measure of the count of monthly readmissions for hyperbilirubinemia decreased by 59% to 2.4 (Fig. 2A). The rate of monthly readmissions decreased from 1.4% to 0.6% during the study period (Fig. 2B). For both the count of readmissions and the rate of readmissions, improvement was demonstrated in January 2023 and sustained for the following 14 months.

**Fig. 3.**
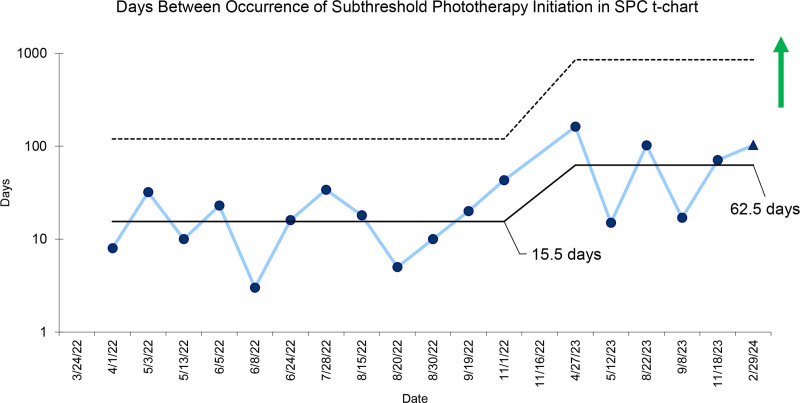
SPC t-chart of days between the occurrence of subthreshold phototherapy initiation at a pediatric hospital, defined as initiating phototherapy at ≥0.3 mg/dL below the AAP threshold. The mean days between the occurrence of subthreshold phototherapy initiation increased from 15.5 to 62.5 days. UCL, upper control limit.

**Fig. 4.**
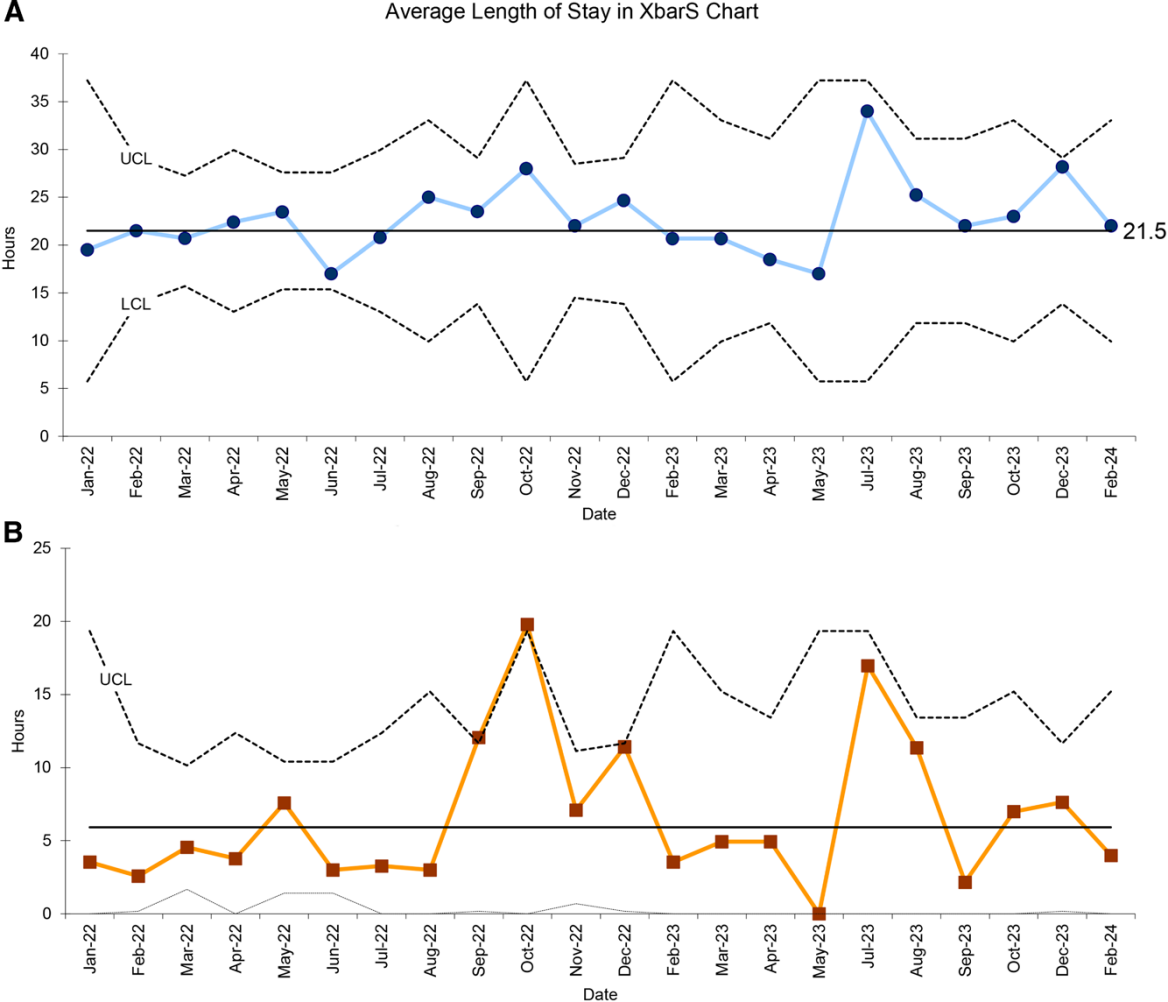
Length of stay. A, Xbar chart of the average length of stay in hours at pediatric hospital for hyperbilirubinemia readmissions. B, S chart. LCL, lower control limit; UCL, upper control limit.

A significant increase in the days between occurrence of subthreshold therapy initiation was observed, increasing from a mean of 15.5 to 62.5 days (Fig. 3). In other words, in the first study period, subthreshold phototherapy was begun every 15.5 days on average. In contrast, subthreshold phototherapy was started much less frequently in the second study period, at an average of every 62.5 days. The balancing measure of average hospital LOS was unchanged at 21.5 hours (Fig. 4).

## DISCUSSION

This QI initiative successfully aligned care across birthing and tertiary care pediatric hospitals with the 2022 AAP CPG for newborn hyperbilirubinemia. Despite variation in readmission seen after the release of the 2022 CPG at other institutions,^19,20^ our institution’s adoption was associated with a significant decrease in readmission for newborn hyperbilirubinemia. Additionally, there was a substantial improvement in the occurrence of subthreshold phototherapy initiation.

Our health system model of collaborative partner hospitals is unique as much of the work to prevent readmissions occurred within a birthing hospital separate from the readmission pediatric hospital. Prior QI work in multiple hospitals across an integrated health system succeeded with multifaceted interventions^21^ and guidelines in standardizing quality care across multihospital health systems.^22^ However, our partner birthing and pediatric hospitals exist in separate health systems with different stakeholders, independent EHRs, and unique provider workflow, leading to distinct challenges in creating hospital-specific interventions. To address these challenges, stakeholders from each hospital collaborated before and throughout the implementation phase to align efforts, create resources, and share results. With interventions occurring in 2 partner hospitals simultaneously, it is difficult to ascertain which intervention had the most significant impact on the outcome measure of monthly readmissions. The timing of the shift in readmissions may be due to the change in the phototherapy threshold in the new AAP CPG after adequate provider education and provider adoption of the new guidelines.

Our team made additional changes to ensure sustainability. Systematic supports were designed to encourage adherence to the new guidelines, including updated note templates and creating a clinical pathway linked to our updated pediatric hospital guidelines. Access to point-of-care guidelines via the EHR has benefitted provider adherence.^23^ Given that almost 2 decades had elapsed since the revision of the 2004 CPG, a substantial focus was dedicated to updating care team knowledge by disseminating the key changes in the 2022 CPG. Education for general pediatrics, emergency medicine, and hospital medicine was important to identify and support newborns qualifying for home phototherapy. However, prior studies have found variation in provider knowledge and adherence to the AAP hyperbilirubinemia CPG,^24,25^ and education interventions alone have a low level of reliability for change.^26^ Therefore, in addition to care team education, ongoing audits were done to provide individual-level education about changes in the new CPG. Formal data collection was completed in February 2024; however, our team continued to monitor guideline adherence through spot audits using a convenience sample through July 2024. The improvement in days between the occurrence of subthreshold phototherapy initiation may reflect the successful implementation of this decision support and education which improves provider adherence.

One ongoing improvement area was the paucity of G6PD screening on newborns readmitted with phototherapy, even those with noted concern for DAT-negative hemolysis. G6PD deficiency is the most common red blood cell enzyme disorder in the world^27^ and is significantly associated with neonatal hyperbilirubinemia, phototherapy, and kernicterus.^28,29^ Racial disparities exist with a disproportionate incidence of kernicterus in Black newborns.^30^ Most newborns with kernicterus secondary to G6PD-deficiency are readmitted from home.^28,31^ These readmissions represent an important focus for an equitable reduction in preventable kernicterus through QI efforts. Future efforts for our improvement team will focus on increasing provider awareness of G6PD deficiency prevalence and considering EHR mechanisms to improve the identification of high-risk neonates.

Of note, LOS for readmitted newborns remained unchanged in our pediatric hospital at 21.5 hours. Similarly, a recent study noted only a 1-hour increase in LOS without a change in the duration of phototherapy with the implementation of the 2022 CPG.^19^ Despite our unchanged LOS, there still may be local opportunities to decrease LOS. For example, administering phototherapy is more efficient in an emergency department observation unit than an inpatient unit.^32^ Furthermore, the 2022 CPG included specific recommendations on obtaining rebound hyperbilirubinemia levels, which may be an opportunity for local improvement, as we saw a trend toward increased inappropriate rebound bilirubin level obtainment in the implementation period. One area for future intervention may be to decrease inappropriate rebound hyperbilirubinemia levels occurring in the inpatient setting, which likely contributes to LOS as those patients remain admitted until follow-up laboratory data are drawn and resulted.

This QI study was performed at partner hospitals in the United States; therefore, results may not be generalizable to other hospital settings or globally to low- or middle-income countries with a higher burden of severe hyperbilirubinemia, higher risk of kernicterus and varied resources.^33,34^ Our sample size was small, although this may represent the proportion of children receiving care at freestanding children’s hospitals.^35^ Our interventions occurred at 2 partner hospitals in separate health systems, sometimes simultaneously, making it more difficult to analyze which interventions impacted the readmission rate most. However, this timeline represents the pragmatic approach to QI interventions, particularly as the timing of EHR interventions can be challenging to predict. Some readmissions for phototherapy may have occurred at community hospital sites outside of our pediatric hospital. However, readmission locations would be stable across the baseline and intervention periods, given no changes in local health insurance and hospital affiliations during the study period.

## CONCLUDING SUMMARY

Using the model for improvement, this QI initiative successfully implemented the 2022 AAP CPG for newborn hyperbilirubinemia across partner birthing and pediatric hospitals. This collaborative QI work significantly reduced monthly readmissions for phototherapy via care team education, systemwide EHR updates, and process improvements. Future work will include improving the screening process for G6PD.

## ACKNOWLEDGMENT

The authors are grateful to Cars Curing Kids for supporting this QI initiative.
